# Evaluation of Routine Microscopy Performance for Malaria Diagnosis at Three Different Health Centers in Brazzaville, Republic of Congo

**DOI:** 10.1155/2018/4914358

**Published:** 2018-09-02

**Authors:** Pembe Issamou Mayengue, Dezi Kouhounina Batsimba, Louis Régis Dossou-Yovo, Roch Fabien Niama, Lucette Macosso, Brice Pembet Singana, Igor Louzolo, Nadia Claricelle Bongolo Loukabou, Géril Sekangue Obili, Simon Charles Kobawila, Henri Joseph Parra

**Affiliations:** ^1^Faculté des Sciences et Techniques, Université Marien Ngouabi, BP 69 Brazzaville, Congo; ^2^Laboratoire National de Santé Publique, BP 120 Brazzaville, Congo; ^3^Ecole Normale Supérieure, Université Marien Ngouabi, BP 69 Brazzaville, Congo; ^4^Centre Hospitalier Universitaire de Brazzaville, BP 1846, Congo

## Abstract

**Background:**

In Republic of Congo, malaria diagnosis still widely relies on microscopy. We aimed to evaluate the performance of routine microscopy for malaria diagnosis at three different health centers in Brazzaville.

**Methods:**

A total of 259, 416, and 131 patients with clinical signs of uncomplicated malaria were enrolled at the Hôpital de Mfilou, Centre de Santé Intégré “Maman Mboualé,” and Laboratoire National de Santé Publique, respectively. Two thick blood smears were prepared for each patient, the first being examined by routine microscopists and the second by expert.

**Results:**

At the Hôpital de Mfilou, sensitivity was 62.1% and specificity was 67.3%. Positive and negative predictive values were 55.6% and 72.9%, respectively. At the Centre de Santé Intégré “Maman Mboualé,” sensitivity was 94.2% and specificity was 33.6%. Positive and negative predictive values were 50% and 89.1%, respectively. At the Laboratoire National de Santé Publique, sensitivity and specificity were high with 91.7% and 94.9%, respectively. Positive and negative predictive values were 64.7% and 99.1%, respectively.

**Conclusion:**

The performance of routine malaria microscopy in Brazzaville remains inaccurate with large variations among different health centers. Therefore, repeated training including supervision and evaluation would improve routine malaria diagnosis for better management of malaria in Brazzaville, the Republic of Congo.

## 1. Introduction

Malaria remains a public health problem in the majority of countries in sub-Saharan Africa. Global morbidity and mortality are estimated at 212 million malaria cases and 429,000 deaths attributable to malaria, respectively, in 2015, with 70% of all malaria deaths occurring among children under the age of five. Approximately 90% of malaria cases and 92% of deaths occurred in the WHO African Region. Thirteen countries, mainly in sub-Saharan Africa, accounted for 76% of malaria cases and 75% deaths globally [1].

In the Republic of Congo, where malaria is still the leading cause of attendance in health facilities, the high level of resistance of* Plasmodium falciparum* to chloroquine and the inefficacy of sulphadoxine-pyrimethamine and amodiaquine either singly or in combination for the treatment of uncomplicated malaria have been well documented [2–5]. Thus, in 2006, the Republic of Congo has changed its antimalarial drug policy for treating uncomplicated malaria to artemisinin-combination therapies [6]. To avoid the threat of potential emergence of drug resistance, accurate diagnosis strategies for malaria, as well as, prompt case management are warranted. Therefore, the World Health Organization (WHO) has recommended a parasitological test to all patients with suspected uncomplicated malaria before initiating antimalarial treatment [7]. Laboratory confirmation of malaria with microscopy or a rapid diagnostic test (RDT) will help prevent unnecessary treatment with expensive artemisinin-combination therapies (ACTs) and the development of drug resistance and will also allow rapid and correct management of other febrile illnesses [8–11].

In the Republic of Congo, diagnosis of malaria is still widely based on microscopy in the majority of health facilities, and confirmatory testing rate with RDTs remains low. A study conducted in Brazzaville has shown that utility of RDTs as a diagnostic tool was poor, due to lack of experience of staff and their lacking availability [12]. This observation has been also supported by the WHO showing no use of RDT tests in 2013 and 2015 in the Republic of Congo [1], although microscopy has been the primary method and remains the gold standard for malaria diagnosis in clinical routine, with rather poor quality mainly at primary healthcare level, thus requiring supervision and training activities.

Over the last five years the Ministry of Health has initiated the “PARAMED” project which aims at capacity building of laboratory technicians was involved in routine malaria microscopy. First, individual microscopists from various healthcare sites were selected and trained in malaria light microsocpy. Subsequently, after their returning to their original health-facility laboratories they would know how to perform microscopy and spread relevant knowledge to their peers. However, the impacts of these trainings are not well documented. Consequently, little is known about the actual performance of malaria routine microscopy in different healthcare level facilities in Brazzaville. Thus, the present study aimed at evaluating the performance of routine microscopy for malaria diagnosis at three different health centers in Brazzaville, the Republic of Congo.

## 2. Methods

### 2.1. Study Areas

Brazzaville, the political capital, hosts 38% (1 642 105 inhabitants) of the total population of the Republic of Congo, estimated at 4 312 715 inhabitants. With the population expansion due to urbanization, Brazzaville is now divided into nine districts: Bacongo, Makelekele, Poto-Poto, Moungali, Ouenze, Talangaï, Mfilou, Madibou, and Djiri. The present study was conducted in three centers: Centre de Santé Intégré (CSI) “Maman Mboualé” located in the district of Talangaï, in the north part of city, Hôpital de Mfilou located in the district of Mfilou, in the south part of the city, and the Laboratoire National de Santé Publique (LNSP), the national reference laboratory located, in the center part of city, in the district of Poto-Poto.

Malaria transmission in the study areas varies from low and moderate to intense with meso-hyper-to perennial endemicity. Malaria infection is primarily due to* P. falciparum*. Two rainy seasons are observed each year with the main one during the months of February to May and a short one from October to December [12]. Instead of their location, the malaria transmission variation, and their ability to receive many patients from all socioeconomic status requiring a large number of microscopits, the CSI “Maman Mboualé” and Hôpital de Mfilou have been also selected based on the participation of 4 microscopists technicians from each center in PARAMED training, who were also present in their centers during the study. However, since the training was done at the LNSP, all miscroscopists from this laboratory were trained.

The quality control of microscopy reading was performed at the laboratory of the Centre Hospitalier Universitaire de Brazzaville (CHU), which is the big referral hospital with a reference laboratory in Brazzaville. Expert reading was performed by the head of the laboratory, who was also trainer in PARAMED, with the urge experience on parasitology and blinded from the results obtained in all three study health centers.

### 2.2. Ethical Clearance and Site Preparation

The study was approved by the institutional “Comité d'Ethique de la Recherche en Sciences de la Santé” (N° 032/CERSSA-2015). In the early project stage, first, a meeting with the site actors including the head of each laboratory and microscopists was organized for the purpose of presenting the project objectives, methodology, and expected results, as well as to obtain microscopists consent. Second, a meeting was held in the various study sites, assuring the availability of material for thick blood smears preparation during the study execution. Also, training was provided ensuring the standardization of thick blood preparation and parasite density assessments in all sites.

### 2.3. Study Population, Blood Samples, and Data Collection

From May 2015 to May 2016, patients with clinical signs of uncomplicated malaria, presenting at the laboratory of one of the three study sites, were invited to participate in this study. Exclusion criteria comprised pregnancy, severe malaria, or other severe illness as judged by the attending physician.

A number of representative patients to be included each month, per week, and per day have been estimated by the statistician taking into account the proportion of malaria reported in each health center, one year before starting the study. In sample size calculations, using a power of 80% and a significance level of 5%, the SCHWARZ method yielded a minimum number of 310, 200, and 100 patients to be recruited at the CSI “Maman Mboualé,” Hôpital de Mfilou, and the LNSP, respectively. Recruited patients were randomly selected from Monday to Friday. At the CSI “Maman Mboualé,” a minimum of 7 patients were recruited per week, with at least one patient per day, whereas at the Hôpital de Mfilou the minimum number was 5 patients per week, with one per day. For the LNSP, 3 patients were enough, with one included on Monday, on Wednesday, and on Friday. After informed consent was obtained, records were made on patient demographics, fever or history of fever in the last 48 hours, other signs of malaria, provenance, and previous antimalarial drugs intake used of bed net treated. The axillary temperature was taken for fever confirmation.

At each study site, two thick blood smears were prepared for each patient, with one being read immediately to inform the patient of the respective result. Before reading, thick blood smears were dried and stained with 10% Giemsa solution (Sigma Chemical, Sigma Aldrich ChemieGmbh, Taufkirchen, Germany) in pH 7.2, for approximately 10 min. The stain was gently washed away by adding drops of clean water and the slide was completely dried before examination. Thick blood smears were assessed by micrsocopists until 200 leucocytes had been counted. Parasite density was calculated for each patient assuming an average of 8000 leucocytes per *μ*l of blood using the proposed method of the WHO [13]. Individual diagnostic result was given to each patient and advised to meet the prescribers for possible antimalarial chemotherapy. The second uncolored thick blood smear was transferred to the CHU for microscopy quality control. At the CHU, also the Giemsa for sample staining was provided.

### 2.4. Data Analysis

Data were entered and verified in Microsoft Excel (Microsoft Corp., Seattle, USA) and validated in EpiInfo for Windows version 3.5.1. Data were analyzed using the SPSS 16.0 for Windows (Inc., Chicago, USA). Age was expressed as median (range), while categorical variables were expressed as percentages. Correlation coefficients were used to compare parasites densities from health centers and quality control expert. The Bayesian theory was used to estimate the sensitivity, specificity, positive predictive value, and negative predictive value as described by Joseph et al. [14]. 95% confidences intervals (95% CI) were generated for parameters mentioned above. Statistical significance of performance differences between health center microscopists and quality control expert was evaluated using McNemar's test. Statistical significance level was set at p<0.05.

## 3. Results

### 3.1. Characteristics of Febrile Patients with Symptoms of Malaria

A total of 259, 416, and 131 patients with suspected malaria were enrolled at the Hôpital de Mfilou, CSI “Maman Mboualé,” and the LNSP, respectively.

Out of 259 patients enrolled at the Hôpital de Mfilou, gender and age were recorded for 257 of them, with 131 being female and 126 male. Thirty-eight patients had an age below 5 years. With regards to CSI “Maman Mboualé,” out of the 416 recruited patients, 410 had records on gender with 207 and 203 being female and male, respectively, and 99 patients were children under 5 years of age (Table 1).

Among 131 patients recruited at the LNSP, 68 and 63 were female and male, respectively, and all of them had an age above 5 years.

### 3.2. Slide Positivity and Quality Control of Three Health Centers in Brazzaville

Of the 259 slides collected at the Hôpital de Mfilou, 115 (44.4%) were reported positive by routine microscopists, whereas 103 (39.8%) slides were positive by the quality control expert. Of the 144 slides that were negative by the routine microscopy, 39 were identified positive by the quality control expert. False positive proportions (51/115; 44.3%) and false negative proportions (39/144; 27.1%) were not statistically significant (*p *= 0.240). Thus, the performance at the Hôpital de Mfilou in terms of sensitivity and specificity was 62.1% (CI: 52.5% -70.9%) and 67.3% (CI: 59.6% -74.2%), respectively. Positive and negative predictive values were 55.6% (CI 46.5% - 64.4%) and 72.9% (CI 65.1% - 79.5%), respectively (Table 2).

With regard to CSI “Maman Mboualé,” 324 (77.9%) slides were reported positive by routine microscopists, while only 172 (41.3%) slides were positive by the quality control expert. Out of the 92 slides that were rated negative by the routine microscopists, 10 were revealed positive by the quality control expert (Table 2). At this site, routine microscopists reported significantly more false positive (162/324; 50%) than false negative (10/92; 10.8%) with* p*< 0.001. Routine microscopists had a performance of high sensitivity at 94.2% (CI: 89. 6%- 96.8%), but of low specificity at 33.6% (CI: 29.0%- 38.7%). Positive and negative predictive values were 50% (CI: 44. 6%- 55.4%) and 89.1 (81.1%- 93.9%), respectively.

At the LNSP, 17 (12.98%) out of 131 slides were reported positive by the routine microscopists, whereas 12 (9.2%) were rated positive by the quality control expert. Out of the 114 slides that were rated negative by routine microscopists, only one was revealed positive by the quality control expert (Table 2). However, the microscopists at this site reported also more false positives (6/17; 35.3%) than false negatives (1/114; 0.9%) with* p*< 0.001.

Both sensitivity and specificity were high at this study site with 91.7% (64.6% -98.5%) and 94.9% (89.4%-97.7%), respectively. Positive and negative predictive values were 64.7% (CI: 41.3%- 82.7%) and 99.1 (95.2%- 99.8%), respectively (Table 2).

### 3.3. Identification of Plasmodium Species

*P. falciparum *was the only species identified in all positive slides confirmed by the quality control expert. However,* P. malariae* was detected by routine microscopists in one and two slides of 64 and 162* P. falciparum *confirmed positives slides from Hôpital de Mfilou and CSI “Maman Mboualé,” respectively. At the LNSP, out of 11 confirmed positive slides, one slide was positive for* Plasmodium ovale* by the routine microscopists.

### 3.4. Evaluation of Parasite Densities at Different Sites

**Figure 1:**
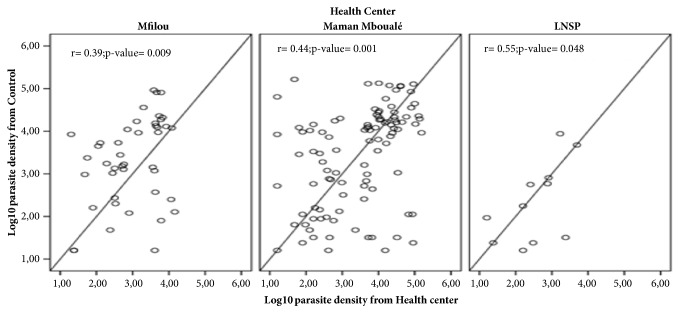
Comparison of parasites densities between different sites and control.

All three study sites assessed parasite density different comparatively to the quality control expert (Figure 1). However, the differences from the quality control expert were more pronounced at the CSI “Maman Mboualé” (*p*= 0.001) and Hôpital de Mfilou (*p=* 0.009) compared to that with the LNSP (*p*= 0.048).

## 4. Discussion

This study demonstrates the inaccurate performance of routine microscopy in the three health centers compared to expert microscopy, indicating poor performance, where large quantities of blood smear examination and permanent involvement of several microscopists were necessary.

Both sensitivity and specificity were significantly higher at the LNSP, which is the reference laboratory at the national level. This performance may be explained by the fact that the LNSP is the national site for microscopist training and as such having qualified trainers and technicians who are also working in parasitological diagnostic routine. Other factors that contribute to this performance were favorable quality of equipment and the relative low number of slides prepared ranging from one to ten, related to low malaria transmission as it was observed in Kenya [15]. However, the relative difference on estimation of parasite density as well as the trend of species misclassification requires training and improvement knowledge on the parasite for better management of malaria cases at this site.

In contrast to LNSP, different performances have been found at the Hôpital de Mfilou and at the CSI “Maman Mboualé.” The lower sensitivity and specificity of routine microscopy were observed at the Hôpital de Mfilou and the CSI “Maman Mboualé.” Both over- and underdiagnoses of malaria are common in malaria-endemic African countries such as Tanzania, Uganda, Burkina Faso, Kenya, and Côte d'Ivoire [10, 16–19]. These lower performances have potential clinical implications, with low sensitivity implying many underdiagnosed malaria cases with no safe and appropriate treatment. Low specificity leads to overdiagnosis with overtreatment and unnecessary prescription of antimalarials, as well as underdiagnosis and undertreatment of real causes of actual symptoms [10, 15, 20, 21]. Plasmodial species were also confused in these two sites and parasite density estimation was largely different from the expert's assessment. The observed phenomena are a worrying situation in these two health centers, receiving a high amount of suspected malaria patients of all age groups, including children under 5 years of age, the population most vulnerable to severe and potentially fatal malaria complications. Additionally, due to the lack of recent entomological data in these study sites, malaria burden is mostly estimated based on the proportion of diagnosed cases. Therefore, misdiagnosis and resulting biased interpretations of in reality-different epidemiological patterns on malaria may lead to biased reports on the local burden of malaria and to biased evaluations of various malaria control intervention programs. Several factors may justify the poor performance of routine microscopy in these sites including the large number of thick blood smears to read in daily routine, lack of skilled microscopists, supervision, and maintenance of infrastructure mainly at the CSI “Maman Mboualé,” and lack of regular quality control. Although four selected microscopists from each of these two sites had recently participated in the PARAMED training, the daily work in these two centers was also done by several nonselected microscopists. However, the obtained results are an indicator of the lack of supervision after training as well as low impact of spread of acquired knowledge to others microscopists working permanently at these sites. Training can improve the capacity of individual microscopists; but, when they return to respective laboratories, they often face many challenges such as equipment and heavy workloads. These challenges can contribute to the marginal improvements in the performance observed after training [15].

The PARAMED training lasted only 1 month with many modules. The short duration of training of microscopists may also impact acquired knowledge. Therefore, permanent training including laboratory supervision as well as improvement of infrastructure quality and implementation of internal and/or external quality control are needed for achieving improvement of malaria diagnosis by microscopy as observed in Angola [22]. The other alternative should be the introduction of RDTs in malaria diagnosis procedure and their systematic use in addition to microscopy in these sites. However study conducted by Ntoumi et al. [12] has shown that the RDTs are not largely use mainly in Brazzaville.

The responsibility of the National Malaria Control Program in the Republic of Congo may be in increasing training on the use of RDTs and implementing their systematic use in the algorithm of malaria diagnosis in the country.

By considering the proportion of positive slides determined by the expert reader, the prevalence of malaria was estimated at 39.77%, 41.34%, and 9.16% at the Hôpital de Mfilou, the CSI “Maman Mboualé,” and the LNSP, respectively. Interestingly, malaria prevalence did not differ between the northern and the southern health centers; however these prevalence cases were high compared to those found previously in the north and south part of Brazzaville [12, 23]. This difference may be explained by the fact that the current study was conducted during one year including all seasons. Additionally, the lack of distribution of malaria bed nets treated since 2013 may have an impact on the increasing incidence of the disease. The different situation observed at the LNSP is probably due to a small number of suspected malaria patients presenting at the laboratory with the majority of patients being adults. Extensive repeated studies in different sites including other departments are needed to provide the actual picture of malaria prevalence in the Republic of Congo.

## 5. Conclusion

This study showed that the performance of routine malaria microscopy in Brazzaville remains substandard between different health centers. Therefore, repeated training including supervision and evaluation at the national level as well as implementation and use of RDTs would improve routine malaria diagnosis for better management and control of malaria in Brazzaville.

## Figures and Tables

**Table 1:** Characteristics of patients.

Characteristics	Hôpital de Mfilou	CSI “Maman Mboualé”	LNSP
Total numbers	259	416	131
Gender (F/M)	131/126	207/203	68/63
Median age (years)	26 (0.5-84)	10 (0.5-75)	42 (11 - 74)
Groups of age (n, %)			
< 5 years	38 (14.8)	99 (23.8)	0 (0.0)
≥ 5 years	219 (85.2)	317 (76.2)	131(100)

**Table 2:** Slide positivity and quality control of three health centers in Brazzaville.

Site		Quality control		Sensitivity	Specificity	PPV	NPV
		Positive	Negative	% (95% CI)	% (95% CI)	% (95% CI)	% (95% CI)
Hôpital de Mfilou	Positive	64	51	62.1 (52.5-70.9)	67.3 (59.6-74.2)	55.6 (46.5-64.4)	72.9 (65.1-79.5)
	Negative	39	105				
CSI Maman Mboualé	Positive	162	162	94.2 (89.6-96.8)	33.6 (29.0-38.7)	50.0 (44.6-55.4)	89.1 (81.1-93.9)
	Negative	10	82				
LNSP	Positive	11	6	91.7 (64.6-98.5)	94.9 (89.4 -97.7)	64.7 (41.3-82.7)	99.1 (95.2-99.8)
	Negative	1	113				

## Data Availability

The data used to support the findings of this study are available from the corresponding author upon request.
